# Telephone peer recruitment and interviewing during a respondent-driven sampling (RDS) survey: feasibility and field experience from the first phone-based RDS survey among men who have sex with men in Côte d’Ivoire

**DOI:** 10.1186/s12874-021-01208-x

**Published:** 2021-02-05

**Authors:** Maxime Inghels, Arsène Kra Kouassi, Serge Niangoran, Anne Bekelynck, Séverine Carillon, Lazare Sika, Mariatou Koné, Christine Danel, Annabel Desgrées du Loû, Joseph Larmarange, for the research team ANRS 12323 DOD-CI, Nelly ASSOUMOU, Anne BEKELYNCK, Christine DANEL, Mohamed DOUMBIA, Mariatou KONE, Alexis KOUADIO, Arsène Kra Kouassi, Serge NIANGORAN, Honoré OUANTCHI, Lazare SIKA, Séverine CARILLON, Maxime INGHELS, Joseph LARMARANGE

**Affiliations:** 1 Lincoln International Institute for Rural Health, University of Lincoln, Brayford Pool, Lincoln, Lincolnshire LN6 7TS UK; 2 Centre Population et Développement (UMR 196 Paris Descartes – IRD), SageSud (ERL INSERM 1244), Institut de Recherche pour le Développement, Paris, France; 3 Programme PAC-CI/ANRS, Centre Hospitalier Universitaire de Treichville, Abidjan, Côte d’Ivoire; 4 École Nationale Supérieure de Statistique et d’Economie Appliquée (ENSEA), Abidjan, Côte d’Ivoire; 5 Institut d’Ethno-Sociologie (IES), Abidjan, Côte d’Ivoire; 6 Centre Inserm 1219, Université de Bordeaux, Bordeaux, France

**Keywords:** Phone-based survey, Respondent-driven sampling, Men who have sex with men, Côte d’Ivoire

## Abstract

**Background:**

Many respondent-driven sampling (RDS) methodologies have been employed to investigate hard-to-reach populations; however, these methodologies present some limits. We describe a minimally investigated RDS methodology in which peer recruitment and interviewing are phone-based. The feasibility of the methodology, field experiences, validity of RDS assumptions and characteristics of the sample obtained are discussed.

**Methods:**

We conducted a phone-based RDS survey among men who have sex with men (MSM) aged 18 or above and living in Côte d’Ivoire. Eight initial MSM across Côte d’Ivoire were selected. Participants were asked to call a hotline to be registered and interviewed by phone. After the participants completed the questionnaire, they were asked to recruit a maximum of 3 MSM from their acquaintances.

**Results:**

During the 9 months of the survey, 576 individuals called the hotline, and 518 MSM completed the questionnaire. The median delay between the invitation to participate and the completion of the questionnaire by peer-recruited MSM was 4 days [IQR: 1–12]. The recruitment process was not constant, with high variation in the number of people who called the hotline during the survey period.

RDS chain convergence to equilibrium was reached within 6 waves for most of the selected variables. For the network size estimation assumption, participants who incorrectly estimated their network size were observed.

Regarding the sample obtained, MSM were recruited from all the regions of Côte d’Ivoire with frequent interregional recruitment; 23.5% of MSM were recruited by someone who does not live in the same region. Compared to the MSM who participated in two other surveys in Côte d’Ivoire, the MSM in our sample were less likely to know about an MSM non-governmental organisation. However, MSM aged 30 years old and above and those with a low level of education were underrepresented in our sample.

**Conclusion:**

We show that phone-based RDS surveys among MSM are feasible in the context of sub-Saharan Africa. Compared to other classical RDS survey methodologies, the phone-based RDS methodology seems to reduce selection bias based on geography and proximity with the MSM community. However, similar to other methodologies, phone-based RDS fails to reach older and less-educated MSM.

**Supplementary Information:**

The online version contains supplementary material available at 10.1186/s12874-021-01208-x.

## Background

Respondent-driven sampling (RDS) is a popular methodology for biological and behavioural surveillance among hard-to-reach or hidden populations [1–4]. This methodology is particularly suitable for the investigation of small communities or those subject to discrimination (who hide their belonging to the community) [5]. Unlike other link-tracing methodologies, such as snowball sampling, RDS allows a representative sample of the targeted population to be obtained with several conditions and assumptions [6, 7]. The collection of information about the link between recruiters and recruited participants and information about the size of each participant’s network during the recruitment process makes it possible to compute sample weights for each individual and adjust the variance estimation.

Two theorems are often used to justify RDS [6]. The first theorem states that as the recruitment process continues (i.e., as the number of recruitment waves increases), the cumulative proportion of an individual’s characteristics in a sample will tend to stabilise around an equilibrium, regardless of the characteristics of the initially recruited subjects (i.e., also referred to as the “seeds”). The larger is the number of successive waves, the more accurate is the real population estimator, regardless of the choice of the first people recruited. The second theorem states that the group of individuals generated by RDS reaches equilibrium at a rapid (i.e., geometric) rate. According to Heckathorn, an approximation to equilibrium is theoretically expected to be reached in fewer than 6 waves with a tolerance of within 2% [6]. In practice, the equilibrium often stabilised between 5 and 10 waves, or even much later in some cases [8].

The validity of RDS estimators depends on six assumptions that were initially stated by Heckathorn [6]. Subsequent enhancements to estimators have been made to reduce the number of assumptions that need to be met [9–11]. For example, when using the Salganik-Heckathorn estimator (i.e., RDS-I), a uniform number of recruited individuals by each participant is not needed because this estimator takes into account the variation in the recruitment differential [11]. When the size of the targeted population is known, the RDS-SS corrects the bias induced by the non-replacement sampling that is not needed [9].

Two strong assumptions remain: (i) *respondents can self-report their network sizes with accuracy,* and (ii) the selection of participants from the recruiter’s network is random. While some authors disagree about the resistance of the RDS method to errors in the participants’ reporting of their network sizes, most authors agree about the large increase in bias and variance with non-random recruitment [12–14]. In practice, validation of a random peer recruitment process is challenging. In most RDS surveys, the recruitment process requires the referred individuals to visit study centres to be interviewed; thus, most recruited individuals live in or near the cities where the study centres are based [3, 4, 15–17]. Moreover, recruited individuals tend to share common characteristics. Several studies have indicated concern regarding the representativeness of the samples obtained in RDS surveys [18, 19]. These surveys suggest that selection bias during the peer recruitment process is responsible for the over- or under-estimation of subpopulations in RDS samples.

Phone-based surveys that are conducted in the West African context are relatively recent. Mobile phone coverage has dramatically increased over the past decade in Côte d’Ivoire, and data indicate that more than 91% of households have at least one mobile phone [20]. Phone-based surveys that are conducted in this context have shown high participation rates compared to those of surveys conducted in the global North [21–23]. These surveys have also been able to reach individuals on a national scale, even though people who live in rural areas tend to be underestimated [21].

Some web-based RDS surveys have also been conducted [24–26]. In certain cases, web-based interviews were completed via phone-based interviews [19]. However, to the best of our knowledge, no RDS survey has adopted a purely phone-based approach, i.e., an approach that consists of interviews conducted by phone and the use of a dedicated hotline for recruitment.

In this paper, we describe the lessons learned from a fully phone-based RDS survey (in which the phone is used for both recruitment and interviewing) that was conducted in Côte d’Ivoire among men who have sex with men (MSM). This subpopulation faces social stigmatisation in Côte d’Ivoire [27] and is considered hard to reach. First, we report field experiences and describe the respondent recruitment process. Second, we discuss the validity of certain assumptions and hypotheses of the RDS methodology in our study context. Third, we compare the sample obtained in our phone-based RDS survey with those obtained in two other MSM surveys that were conducted in the same context but employed a different sampling design.

## Methods

Between 25th April 2018 and 1st February 2019, we conducted a phone-based RDS survey among MSM aged 18 or above who live in Côte d’Ivoire as part of the ANRS 12323 DOD-CI research project. The utilisation of the phone in this survey was motivated by the premise that MSM often rely on phone applications to meet other MSM, as reported by local MSM non-governmental organisations (NGOs). Initially, seven seeds were selected across Côte d’Ivoire. The telephone contact information of each seed was obtained from various MSM NGOs in Côte d’Ivoire. The emphasis was placed on obtaining seeds from various regions of residences and seeds in various proximities to MSM community networks. Because of the slow recruitment process, one additional seed from a region with low recruitment (i.e., north of Côte d’Ivoire) was added in September 2018.

The seeds received a text message that invited them to participate in the survey. None of the text messages mentioned that the survey was MSM-related to avoid any unwanted disclosure of participants’ sexual orientation. In the text message, a toll-free hotline number was provided for individuals who wanted to participate. Two of the three eligibility criteria (i.e., male, aged 18 or above and living in Côte d’Ivoire) were assessed for everyone who called the hotline. People who were eligible because they met two criteria were offered to be interviewed at a convenient time. During the first part of the interview, we collected non-MSM-related data (demographic characteristics and human immunodeficiency virus (HIV) testing practices) and then collected data regarding sexual behaviours. At the end of this section, the third eligibility criterion was assessed by asking participants if they had previously had sex with women only, men only or both women and men.

For the participants who reported ever having sexual intercourse with a man, MSM-related data (e.g., MSM identity perceptions and access to MSM-related health structure) were collected. At the end of the interview, the participants were invited to refer a maximum of 3 other MSM from their acquaintances. If a participant agreed to provide referrals, a text message with a brief introduction of the study (that did not mention that the survey was MSM-related), the referral number to provide to acquaintances and the toll-free hotline number were sent to the participant for recruitment purposes. Participants were asked to call or speak with the MSM who they wanted to refer and send them the introductory text message. A financial incentive of 1500 FCFA (2.5 US dollars) was sent to the recruiting participant using a telephone cash transfer for each recruited participant who completed the questionnaire. No financial incentives were given for participation only or if the participant only referred peers who did not fully complete the questionnaire.

For participants who reported having had sex only with women during the data collection, the questionnaire ended at this section: these individuals were thanked for their participation but were not invited to recruit other participants. These individuals were then excluded from the analysis.

To avoid repeat enrolments, the phone number of every individual who called the hotline was systematically compared with the phone numbers of previous participants. The referral number that was given was also checked during the hotline call to avoid more than three referrals by participants. There was no time limit for referrals.

We initially planned to recruit between 500 MSM and 900 MSM to obtain a sample size comparable to other RDS surveys that were conducted in the subregion [3, 4, 15, 28]. Recruitment was stopped when the number of new recruits became too low to financially maintain the survey.

To allow RDS-weighted calculations, we linked the recruiter to the recruited using their referral number and asked each participant for an estimation of their MSM network size (i.e., “In total, how many men who have sex with men do you know, whether they are friends, partners, or acquaintances?”). Voltz-Heckathorn’s RDS-II estimator was applied for the analysis as it is commonly utilised in the literature and is shown to perform very well when a small sampling fraction is employed [11, 29]. The empirical likelihood interval was calculated using an estimated size of the total MSM population in Côte d’Ivoire of 29,500 individuals based on The Joint United Nations Programme on HIV and AIDS (UNAIDS) estimations [30, 31]. The seeds were kept in the analyses as the bias due to their convenience selection was considered to be negligible [9, 32]. The RDS design of the study was taken into account in the analyses using the RDS package in R [30].

The sociodemographic characteristics of the MSM included in our sample were compared with those of the MSM who participated in the Integrated Biological and Behavioural Surveillance (IBBS) study and CohMSM cohort study [33, 34].

The IBBS surveys aimed to measure HIV prevalence and sexual behaviour among specific high-risk population groups. In Côte d’Ivoire, the last IBBS survey among MSM was conducted from January 2015 to October 2015 among 1301 MSM. The study included 5 RDS surveys that were implemented in 5 Ivorian cities. Three to four seeds were employed to obtain the estimated sample size for each city. The questionnaires were completed face-to-face at a study centre, and peer recruitment was performed using coupons.

CohMSM is an open cohort of MSM who attended sexual health clinic visits with different services, including pre-exposure prophylaxis. The CohMSM study has been conducted since 2015 in four West African countries. In Côte d’Ivoire, participants were recruited mainly in Abidjan via peer educators. Baseline and follow-up visits required the participant to be present in person at the study centre. In this article, we only considered participants who were recruited in Côte d’Ivoire from 2015 to January 2020.

## Results

### Recruitment process

In total, 576 individuals called the hotline number (Table 1). Among these individuals, 39 could not be reached by phone after being registered via the hotline, 2 were not eligible (lived outside Côte d’Ivoire or were younger than 18 years old), and 1 spoke a language that was not spoken by the investigators. Among the remaining 534 individuals, 16 reported never having had sex with a man and were thus excluded from the analysis. In total, 518 MSM were interviewed.

**Table 1:** Results of telephone calls among men who have sex with men, 2018–2019

		n	%
	**Registered phone numbers**	576	100.0
EF	Respondent unable to be reached, no contact after initial registration	20	3.5
EF	Respondent could not be reached after appointment (questionnaire started or not)	10	1.7
EF	Wrong phone number given when making an appointment	9	1.6
OT	Language barrier with the respondent	1	0.2
OT	Respondent was not living in Côte d’Ivoire	1	0.2
OT	Respondent was younger than 18 years old	1	0.2
	**Individuals interviewed**	534	92.6
	–	–	–
	**Individuals interviewed**	534	100.0
OT	Respondent reported never having had sex with a man	16	2.8
EF	Respondent discontinued questionnaire	0	0.0
	**Completed questionnaires**	518	97.2
	–		
	**Overall execution failure rate**(Eligible individuals with off-target persons excluded)	–	7.0

*EF* Execution Failures; *OT* Off Target

**Fig. 1:**
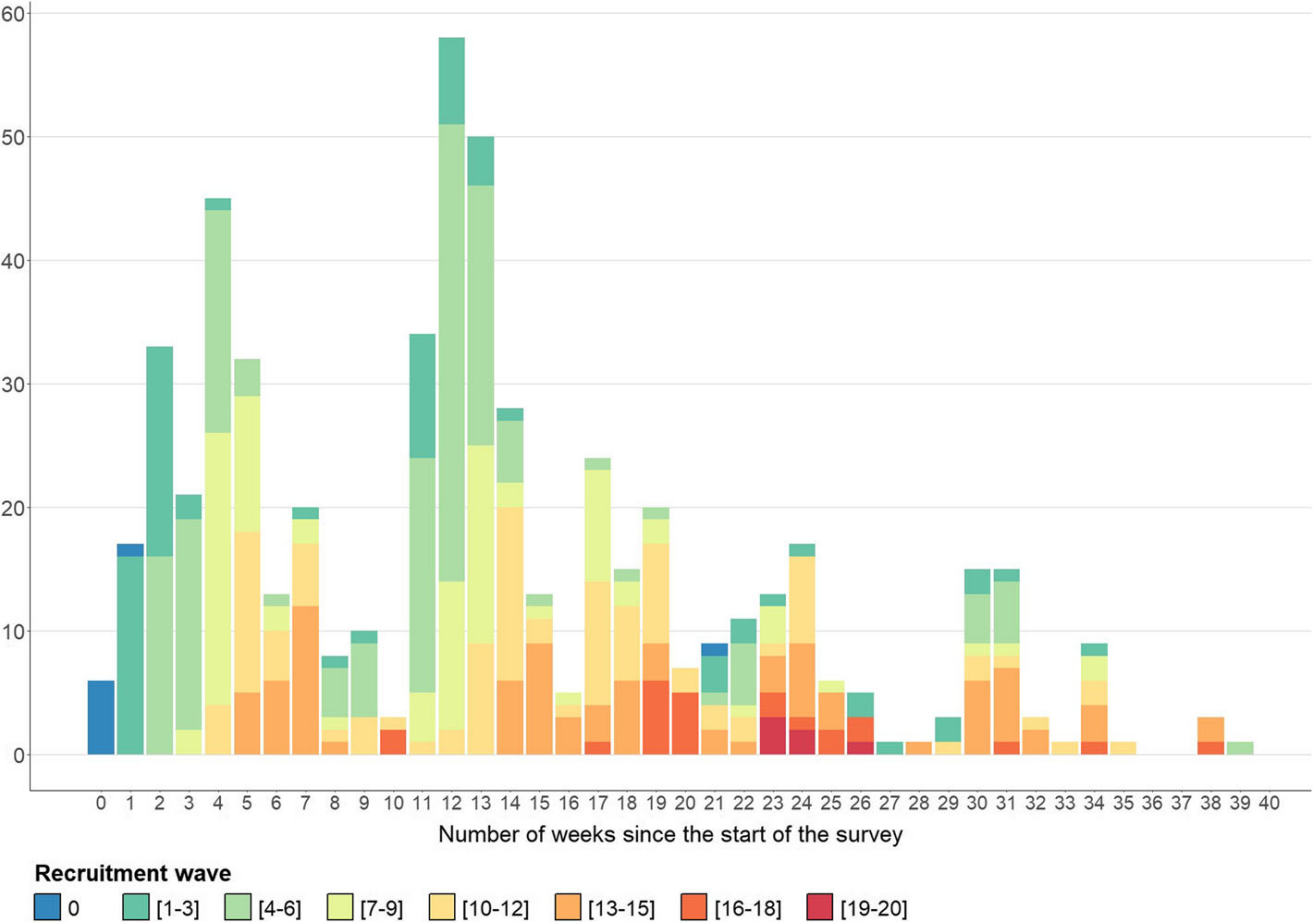
Number of individuals registered (via the hotline) per week and per recruitment wave, 2018–2019 (*n* = 576). Note 1: This figure includes execution failures and non-eligible people who call the hotline. Note 2: Seven seeds were introduced in week 1, and an additional seed was introduced in week 21

Once they completed the questionnaire, the MSM were invited to recruit a maximum of three other MSM; 99.4% of them accepted this offer. The latter totalised the theoretical possibility to refer a maximum of 1530 MSM of their acquaintances, of which 510 (33.1%) led to the referral of MSM who completed the questionnaire. The recruitment process was not uniform over time, with a high variation in the number of people who call the hotline per week (Fig. 1). The 8th seed that was introduced in week 21 had a minor effect on the recruitment process.

**Fig. 2:**
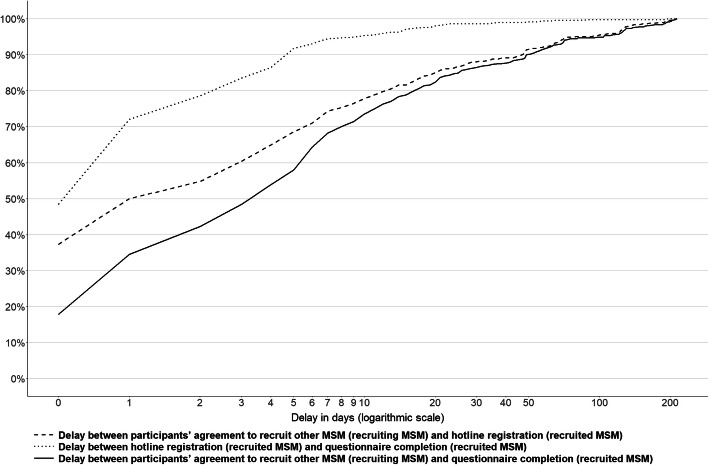
Delay between participants’ agreement to recruit other MSM at the end of the questionnaire and hotline registration of the recruited MSM and participants’ completion of the questionnaire by the recruited MSM, 2018–2019 (*n* = 510). Note 1: The percentage corresponds to the cumulative proportion. Note 2: The eight seeds are excluded

The median delay between the completion of the questionnaire by MSM and the registration of their recruited MSM was 1 day [interquartile range (IQR): 0–8]. The median time between participant registration and questionnaire completion was 1 day [IQR: 0–2]; thus, the median time between the invitation to recruit additional participants and questionnaire completion by recruited MSM was 4 days [IQR: 1–12]. The delay between recruitment and completion was more dependent on the delay in recruiting MSM rather than the delay in completing the questionnaire once MSM had registered via the hotline (Fig. 2).

**Fig. 3:**
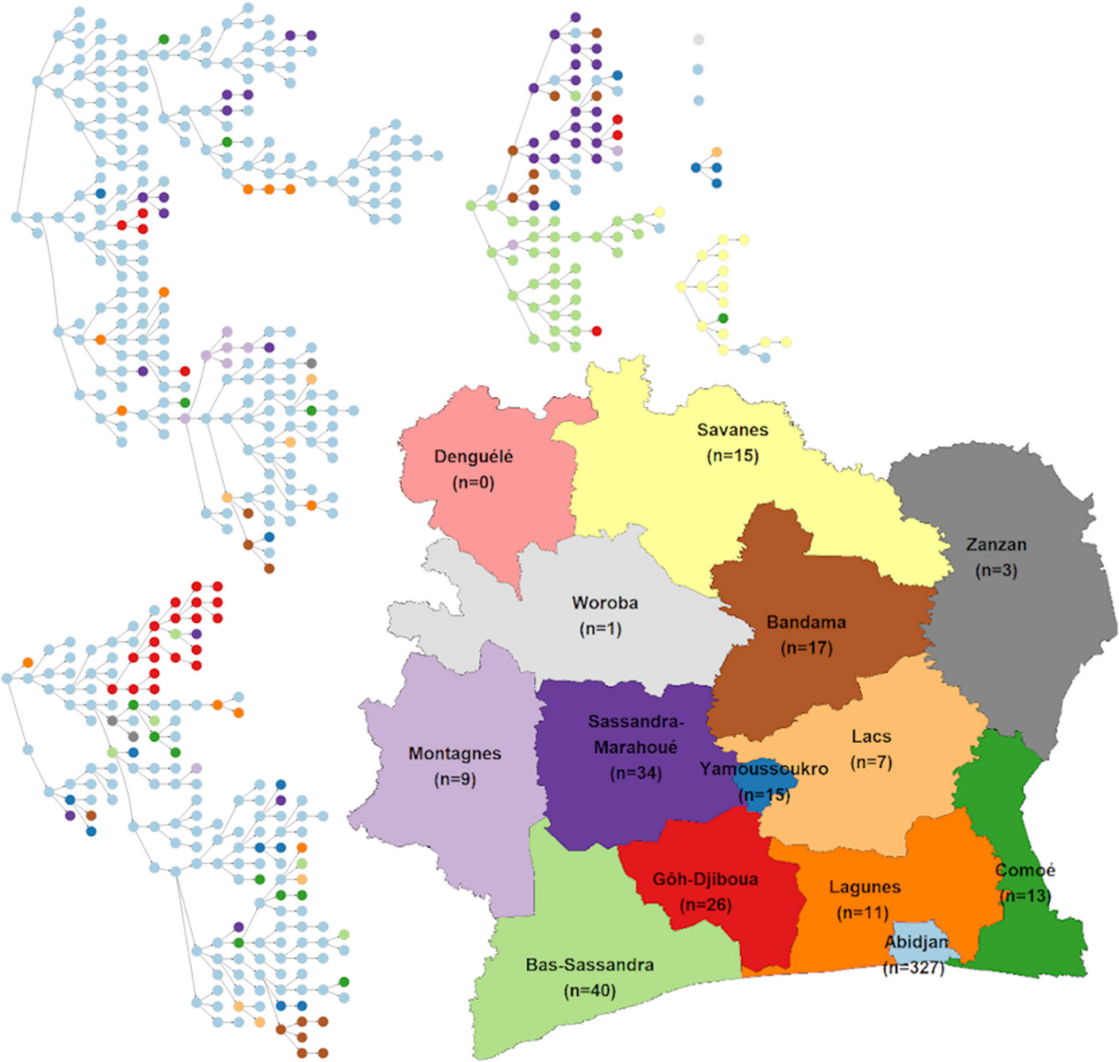
Recruitment networks by region of Côte d’Ivoire, DOD-CI study, 2018 (*n* = 518). Note: The depicted map is original and was not obtained from another source

Four seeds were recruited in the Abidjan region, and three seeds were recruited in the Woroba, Bas-Sassandra and Yamoussoukro regions (Fig. 3). The additional 8th seed was recruited in the Savanes region and led only to 16 peer recruits. Three seeds led to no MSM referrals, and one seed led to only three recruited MSM. The two seeds recruited in Abidjan led to the recruitment of most of our sample (46.3 and 33.4%).

### RDS equilibrium theorems

**Fig. 4:**
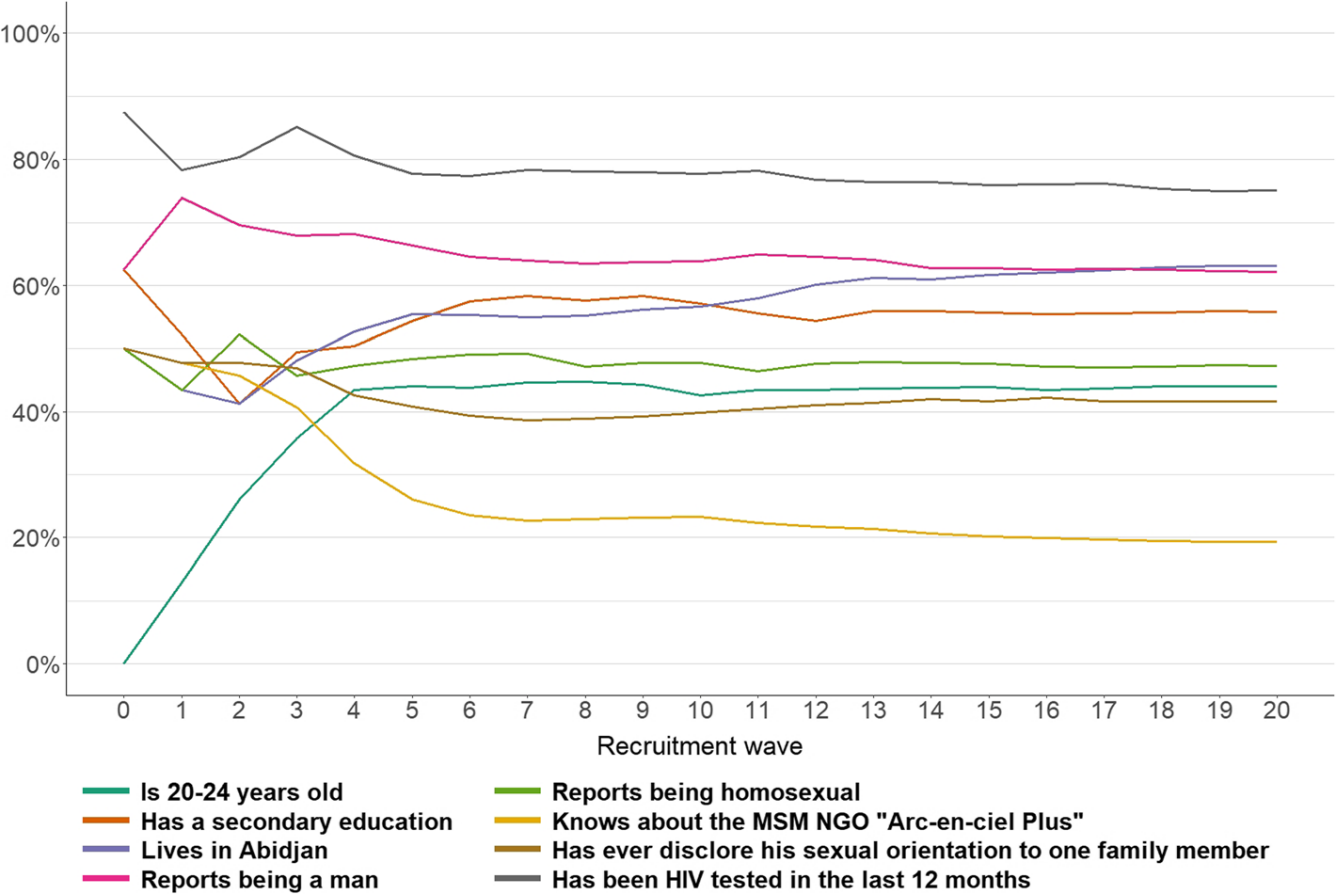
Cumulative distribution of eight indicators by recruitment wave, DOD-CI study, 2018 (n = 518). Note: In this graph, the distribution is cumulative. The proportion of a variable in wave 5 is calculated based on all individuals recruited from wave 0 to wave 5 (inclusive)

As previously mentioned, the two RDS theorems state that equilibrium should be reached at a rapid rate [35]. For 7 of the 8 selected variables, equilibrium was reached in wave 6 (Fig. 4). For one variable (living in Abidjan), a plateau was visible at wave 16, but the cumulative proportion was still slightly increasing over the waves.

### Participant network estimation

Regarding network size, the respondents estimated that they knew a median number of 20 MSM [IQR: 10–50] in their network. Even though it was not possible to verify whether the network size estimation was accurate, errors in participants’ estimation of the size of their networks were observed. Some participants reported inconsistent numbers of MSM in their networks (5 participants and 3 participants reported either no MSM in their networks or fewer MSM in their networks than the number of MSM that they recruited or than the number of their male sexual partners in the last 12 months, respectively). Some participants (6.6%) reported networks of more than 100 MSM (refer to supplemental file 1).

We documented the effect of a correction that was applied to extreme values. When reducing outliers to a more realistic value (values above 100 were reduced to 100), there was a minimal effect on the estimators and their confidence intervals (refer to supplemental file 2).

### Sample characteristics and comparison with the IBBS and CohMSM samples

Most of the MSM recruited in the present DOD-CI sample lived in Abidjan (63.1%), but MSM were recruited across all the regions of Côte d’Ivoire; however, in three of the regions, fewer than 5 MSM were recruited (Table 2). Recruitment across regions was common, as 23.5% of MSM were recruited by someone who did not live in the same region (Fig. 3). Abidjan was more represented in the DOD-CI sample than in the IBBS sample but far less represented than in the CohMSM sample, which was exclusively based in Abidjan.

**Table 2:** Description of characteristics reported by MSM from the DOD-CI (2018), IBBS (2015) and CohMSM (2018) study samples, Côte d’Ivoire

	DOD-CI (*n* = 518)			IBBS (*n* = 1301)			CohMSM (*n* = 274)	
	unweighted		RDS-weighted	unweighted		RDS-weighted		
	n	%	%	n	%	%	n	%
**Age**							*NA = 18*	
< 20	49	**9.5**	**14.3**	154	**11.8**	**14.8**	21	**8.2**
20–24	228	**44.0**	**47.1**	639	**49.1**	**50.4**	111	**43.4**
25–29	169	**32.6**	**35.9**	374	**28.8**	**27.2**	80	**31.3**
> =30	72	**13.9**	**2.7**	134	**10.3**	**7.6**	44	**17.2**
**Level of education**							*NA = 18*	
Primary/none	38	**7.4**	**5.6**	138	**10.6**	**8.7**	26	**10.2**
Secondary	289	**55.8**	**66.1**	810	**62.3**	**69.0**	104	**40.6**
Tertiary	191	**36.9**	**28.3**	353	**27.1**	**22.4**	126	**49.2**
**Self-reported gender**								
Man	322	**62.2**	**65.1**	962	**73.9**	**85.1**	133	**48.5**
Woman	130	**25.1**	**22.2**	339^a^	**26.0**^a^	**14.9**^a^	48	**17.5**
Transgender	66	**12.7**	**12.7**				–	–
Both man and woman	–	–	–	–	–		93	**33.9**
**Sexual orientation**							*NA = 3*	
Homosexual	245	**47.3**	**42.1**	499	**38.4**	**35.1**	115	**42.4**
Bisexual	251	**48.5**	**49.8**	775	**59.6**	**64.0**	145	**53.5**
Heterosexual	22	**4.2**	**8.1**	27	**2.1**	**0.9**	11	**4.1**
**Reported HIV status**								
Never tested	41	**7.9**	**11.1**	259	**19.9**	**29.1**	0	**0.0**
Negative (≤12 months)	345	**66.6**	**60.4**	656	**50.4**	**42.8**	224^b^	**81.8**^b^
Negative (> 12 months)	73	**14.1**	**17.1**	332	**25.5**	**26.1**		
Positive	34	**6.6**	**4.3**	54	**4.2**	**2.0**	49	**17.9**
Do not know	25	**4.8**	**7.8**	–	–	–	1	**0.4**
**Disclosure of sexual orientation to one family member**							NA = 17	
Yes	216	**41.7**	**29.9**	393	**30.2**	*DNA*	140	**54.5**
No	302	**58.3**	**70.1**	908	**69.8**	*DNA*	117	**45.5**
**Knowledge of the MSM NGO “Arc-en-ciel Plus”**								
Yes	100	**19.3**	**10.1**	531	**40.8**	*DNA*	–	–
No	418	**80.7**	**89.9**	770	**59.2**	*DNA*	–	–
**Knowledge of the “Clinique Confiance”**				NA = 200				
Yes	178	**34.4**	**20.7**	645	**58.6**	*DNA*	–	–
No	340	**65.6**	**79.3**	456	**41.4**	*DNA*	–	–
**Region of residence**							NA = 17	
Abidjan	327	**63.1**	**58.3**	351	**27.0**	**10.9**	249	**96.9**
Yamoussoukro	15	**2.9**	**3.7**	250	**19.2**	**18.4**	–	–
Agboville (Lagunes)	11	**2.1**	**1.2**	200	**15.4**	**18.2**	–	–
Bouaké (Bandama)	17	**3.3**	**5.5**	350	**26.9**	**35.0**	–	–
Gagnoa (Gôh-Djiboua)	26	**5.0**	**7.8**	150	**11.5**	**17.5**	–	–
Other regions	122	**23.6**	**23.5**	–	–	–	8	**3.1**

*MSM* Men who have sex with men; *NGO* Non-governmental organisation; *DNA* Data not available

^a^For the IBBS survey, woman and transgender were grouped

^b^For the CohMSM study, participants were only asked if they had a negative test

Age and level of education were quite similar between the DOD-CI sample and the IBBS sample. In CohMSM, MSM were older and had higher education levels.

The percent of MSM who report being HIV-positive in the DOD-CI sample was higher than that in the IBBS sample (4.3% vs 2.0%) but lower than that in the CohMSM sample (17.9%).

Self-reported gender and sexual orientation were different across the three samples. The number of participants who reported a gender other than male was higher in the CohMSM sample (51.5%) than in the DOD-CI sample (34.9%) and IBBS (14.9%) sample. More MSM in the DOD-CI sample than in the other two samples reported being heterosexual (8.1% versus 0.9% in the IBBS sample and 4.1% in the CohMSM sample). The disclosure of sexual orientation to one family member was more frequently reported in the CohMSM study (54.5%), followed by the DOD-CI survey (41.7%, unweighted proportion) and IBBS survey (30.2%, unweighted proportion).

In both the DOD-CI survey and IBBS survey, MSM were asked if they knew about the MSM NGO “Arc-en-ciel Plus”, which is an NGO that is represented mostly in the southeastern regions of Côte d’Ivoire, including Abidjan, and if they knew about the “Clinique Confiance”, which is a health centre dedicated to key populations, such as MSM and sex workers based in Abidjan. Although most MSM who participated in the DOD-CI survey were from Abidjan, they were less likely to report knowing about the NGO “Arc-en-ciel Plus” and “Clinique Confiance” than those in the IBBS study (19.3% versus 40.8 and 34.4% versus 56.8%, respectively; unweighted proportions).

## Discussion

To the best of our knowledge, our survey is the first phone-based RDS survey in which both recruitment and interviewing were conducted by phone in sub-Saharan Africa. Our results showed that the phone-based RDS methodology is feasible among MSM—a population that is considered hard to reach in Côte d’Ivoire because of the stigmatising environment [27]. This methodology, therefore, has the potential to be implemented with other hard-to-reach populations in the sub-Saharan context.

One important result of our study was the high cross-regional recruitment as 23.5% of the MSM included in our sample were recruited by someone who did not live in the same region. RDS chain convergence to equilibrium requires characteristics mixing of sufficient participants, which is rarely met for the region of residence in traditional in-person RDS as chains rarely cross regions in these studies [15, 28, 33]. Therefore, these studies implement separate RDS surveys in each region that is considered, and the results are often aggregated into a pooled estimate. Phone-based RDS could then lead to a better regional representation of the distribution of MSM in the study by facilitating the recruitment of MSM who live far from cities where traditional in-person RDS surveys are conducted.

The use of phone-based peer recruitment and questionnaire interviewing provided significant advantages during the peer recruitment process for both participants and investigators. First, participants could choose the day and time of their interview and did not need to travel, which saved time and transport costs compared to RDS methodologies that require face-to-face interviews. Second, the determination of the eligibility of participants was more convenient and avoided the tasks associated with the management of non-eligible people who visit a study centre, as reported in one survey [36]. Third, the methodology facilitated peer recruitment since only a text message was needed. It was also possible to send reminder text messages for participants who agreed to recruit MSM from their networks and who had not yet recruited new participants—a strategy we applied during the weeks with low recruitment, which led to several additional participants in the survey. Coupon loss was also avoided since it was possible to re-send the referral code to a participant who had lost it. Phone-based RDS avoided potential breaches of anonymity. As participants were not required to be present in person for the interview, unwanted disclosure of sexual orientation was limited as participants could not be seen or recognised by visiting a survey centre.

Despite these advantages, participant recruitment was relatively slow compared to that in the IBBS survey. In 10 months and starting from 13 seeds, the IBBS survey included more than 1301 MSM; in our survey, in 9 months and starting from 8 seeds, we included only 518 MSM. There are two possible explanations for these differences. First, our survey was conducted after several other surveys were conducted in the same community, which may have caused “study fatigue” and decreased interest in participating. Second, prior to the implementation of their survey, researchers of the IBBS organised several gathering events in the different cities where the survey was being conducted, which may have sensitised MSM to participate in the survey. Researchers who perform RDS surveys (phone-based or otherwise) in the future should consider advertising surveys among the MSM networks to hasten the recruitment process. Nevertheless, the characteristics of our recruitment process (i.e., total number of individuals recruited, number of seeds and data collection duration) remained comparable to those of other RDS surveys that were implemented in the West African context [4]. Another significant disadvantage of the phone-based RDS was the inability to collect biological data, which renders this methodology less adapted for disease-related surveillance.

Our results showed that the phone-based RDS methodology seems to satisfy the rapid convergence of RDS chains to equilibrium but still has some limitations regarding the validation of some of the hypotheses, similar to other traditional RDS methodologies.

Consistent with the RDS theorems, equilibrium was reached for most of the variables explored in our survey. With phone-based RDS, similar to other classical RDS methodologies that are based on face-to-face interviews, equilibrium tended to be reached rapidly [8, 17].

The assumption of a random peer recruitment process is a strong hypothesis that is assumed in most RDS surveys but rarely validated [12–14]. By using a phone-based approach, we were likely able to reduce the geographical bias that was induced by in-person referral. However, in the absence of a national reference (population census does not include data on sexual orientation), it is not possible to measure this effect.

Similar to other RDS methodologies and MSM surveys, with our approach, we encountered difficulties reaching older and less educated MSM [3, 15, 16].

Inaccuracy in the estimation of personal network size may be responsible for substantial biases in the estimates [1, 13]. In our survey, some participants reported a very high number of MSM in their networks (above 100), but this finding had a negligible impact on the RDS estimators. It was not possible to assess the accuracy of participants’ network sizes for participants with small networks, which could have led to important biases [13].

In terms of sociodemographic characteristics, the sample that was obtained using phone-based RDS was quite similar to the sample of a previous face-to-face RDS survey and a previously investigated MSM cohort. Some differences were observed, including the proximity to MSM communities and the region of residence. Participants in our sample were also less likely to know about one of the largest Ivorian MSM NGOs, which suggests that phone recruitment could potentially reach MSM who are less engaged in the MSM community. Although our sample was mainly concentrated in Abidjan, a large part of the sample was recruited in other regions of Côte d’Ivoire.

Similar to other surveys that were conducted among MSM in sub-Saharan African countries, our sample was mainly composed of young and educated MSM [15, 16, 25]. Thus, even with a phone-based methodology, which is supposed to reduce selection bias, older and less-educated MSM continue to be underestimated in RDS surveys [15].

Several surveys have investigated the effects of web-based or application (app)-based RDS methodologies on the recruitment process and the characteristics of recruited participants. These studies showed that web-based RDS allowed a faster peer recruitment process than traditional RDS but tended to reach different types of participants [24, 26, 37, 38]. Individuals that were recruited via web-based RDS tend to have a higher socioeconomic status, especially a higher education level, than those recruited via off-line RDS methodologies [24, 37, 38]. Proximity to MSM communities varied according to the survey, with one survey showing fewer gay-identified MSM among those recruited via web-based methodologies in Canada and another survey showing more MSM who identify themselves as homosexual and having sex only with men in China [38, 39]. To our knowledge, no web- or app-based RDS has been implemented in sub-Saharan Africa. In this specific context, we believe that these web-based approaches would tend to induce selection bias since internet access (and thus possession of an email address and access to web applications) remains inequal in African countries [40]. Phone-based RDS could be a more appropriate alternative solution in the context of poor internet access.

RDS methodologies that require the presence of participants continue to be broadly utilised in epidemiological surveillance. Unlike web and phone-based RDS, these methodologies allow the collection of biological data (e.g., blood sample for HIV test). On the other hand, phone-based surveys are usually well adapted to the collection of data on sexual behaviours: several surveys in France and Côte d’Ivoire have shown that people are quite comfortable answering sexuality-related questions by phone [21, 22, 41]. In addition, in our survey, the percentage of people who self-report being HIV-positive was higher than that in the IBBS survey, which suggests a higher acceptance of discussing stigmatising subjects, such as HIV status, by phone in this context.

## Conclusion

We showed that phone-based RDS surveys among MSM are feasible in the context of sub-Saharan Africa, as they have the ability to reach MSM from different geographical regions and some individuals located far from MSM communities and to collect sensitive data, such as data on sexual behaviours. Compared to face-to-face RDS surveys, phone-based RDS surveys still present challenges in reaching older and less-educated MSM.

Phone-based RDS may be a suitable alternative for behavioural surveillance that does not require the collection of biological data from participants.

## Supplementary Information

**Additional file 1.** MSM network size reported by recruitment wave.

**Additional file 2.** Distribution by age, education level and reported sexual orientation with and without outlier correction (for values above 100) of the MSM network size.

## Data Availability

The datasets that were analysed during this are not publicly available as they are the property of the DOD-CI project. Data are, however, available from the authors upon reasonable request and with permission of one of the principal investigators.

## Abbreviations

DNA: Data not available
IBBS: Integrated biological and behavioural surveillance
MSM: Men with have sex with men
NGO: Non-governmental organisation
RDS: Respondent-driven sampling
UNAIDS: Joint United Nations programme on HIV/AIDS
