# Supplementary material for: Telephone peer recruitment and interviewing during a respondent-driven sampling (RDS) survey: feasibility and field experience from the first phone-based RDS survey among men who have sex with men in Côte d’Ivoire
Source: BMC Med Res Methodol. 2021 Feb 5;21:25. doi: 10.1186/s12874-021-01208-x (PMC7866744; doi:10.1186/s12874-021-01208-x)
Supplement: Supplementary file 1 — Additional file 1. MSM network size reported by recruitment wave. [file 12874_2021_1208_MOESM1_ESM.docx]

**Appendix 2. Distribution by age, education level and reported sexual orientation with and without outlier correction (for values strictly above 100) of the MSM network size, DOD-CI study, 2018 (n=518).**

|  | **Without outlier correction** | |  | **With outlier correction** | |
| --- | --- | --- | --- | --- | --- |
|  | **%** | **95% CI** |  | **%** | **95% CI** |
| **Age** | |  |  |  |  |
| 18–19 | 14.3 | [8.3–20.4] |  | 14.3 | [8.2–20.3] |
| 20–24 | 47.0 | [38.9–55.2] |  | 47.0 | [38.8–55.2] |
| 25–34 | 36.0 | [28.5–43.5] |  | 36.1 | [28.6–43.6] |
| 35–44 | 2.3 | [1,3–3.2] |  | 2.3 | [1.3–3.2] |
| 45 and over | 0.4 | [0.1–0.7] |  | 0,4 | [0.2–0.7] |
| **Level of education** | |  |  |  |  |
| None | 1.1 | [0.0–3.0] |  | 1,1 | [0.0–2.9] |
| Primary | 4.5 | [1.1–7.9] |  | 4.5 | [1.1–7.8] |
| Secondary | 66.2 | [58.6–73.7] |  | 66.1 | [58.6–73.7] |
| Tertiary | 28.3 | [21.3–35.4] |  | 28.3 | [21.3–35.4] |
| **Sexual orientation** | | |  |  |  |
| Homosexual | 42.1 | [34.0–50.1] |  | 42.0 | [34.0–50.1] |
| Bisexual | 49.8 | [41.6–57.9] |  | 49.8 | [41.7–58.0] |
| Heterosexual | 8.2 | [4.6–11.7] |  | 8.1 | [4.6–11.7] |

95% CI: 95% Confidence interval

Note: The numbers were calculated according to the RDS-II estimator and rounded to the nearest tenth; therefore, the totals may vary by one unit.
