# Supplementary material for: Telephone peer recruitment and interviewing during a respondent-driven sampling (RDS) survey: feasibility and field experience from the first phone-based RDS survey among men who have sex with men in Côte d’Ivoire
Source: BMC Med Res Methodol. 2021 Feb 5;21:25. doi: 10.1186/s12874-021-01208-x (PMC7866744; doi:10.1186/s12874-021-01208-x)
Supplement: Supplementary file 2 — Additional file 2. Distribution by age, education level and reported sexual orientation with and without outlier correction (for values above 100) of the MSM network size. [file 12874_2021_1208_MOESM2_ESM.docx]

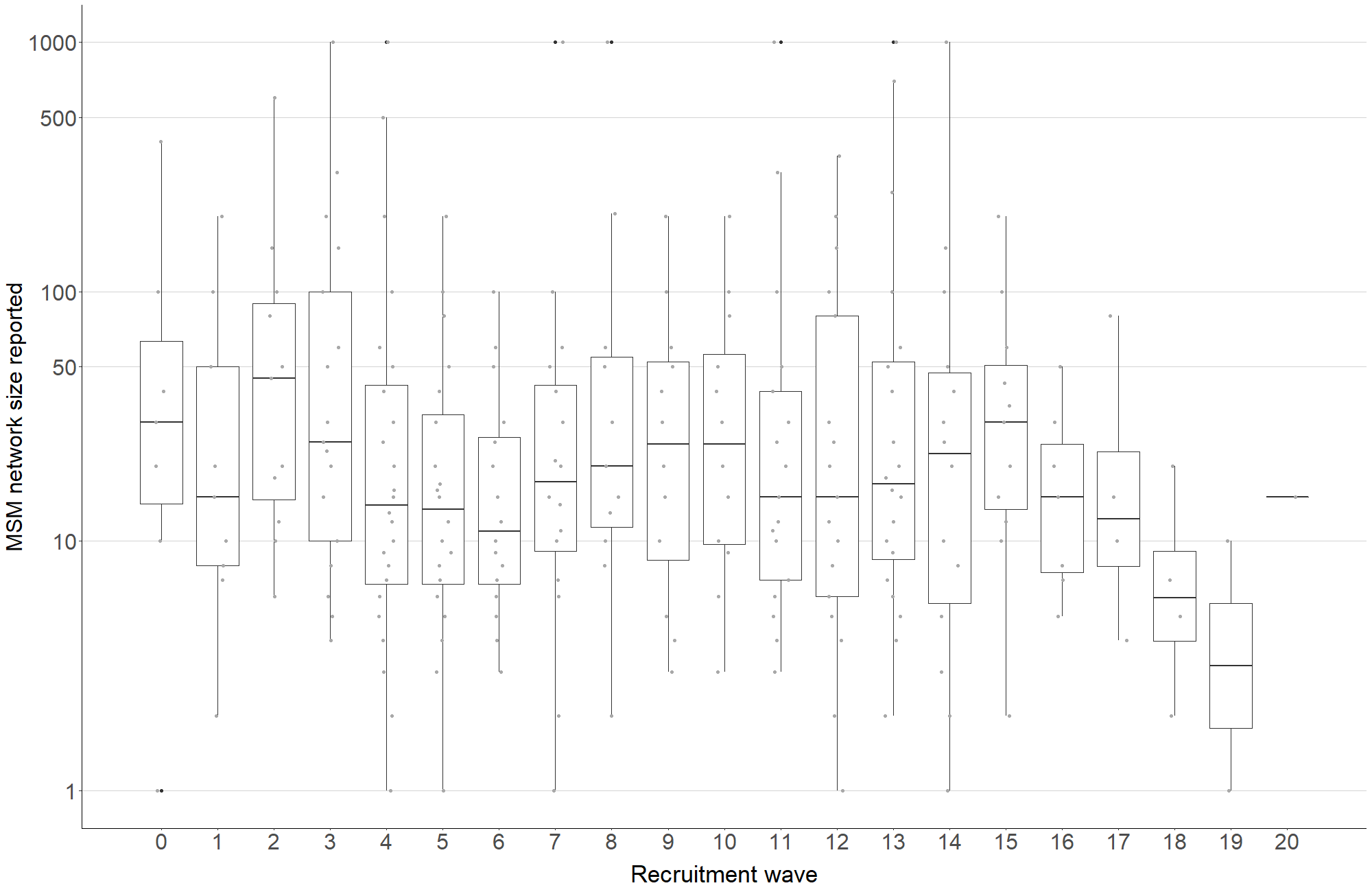
 **Appendix 1. MSM network size reported by recruitment wave, DOD-CI study, 2018 (n=518).**

Note: The MSM network size is described on a logarithmic scale.
